# Determination of Antiepileptics in Biological Samples—A Review

**DOI:** 10.3390/molecules29194679

**Published:** 2024-10-02

**Authors:** João Martinho, Ana Y. Simão, Mário Barroso, Eugenia Gallardo, Tiago Rosado

**Affiliations:** 1 Centro de Investigação em Ciências da Saúde, Faculdade de Ciências da Saúde da Universidade da Beira Interior (CICS-UBI), 6200-506 Covilhã, Portugal; joao.pedro.martinho@ubi.pt (J.M.); anaaysa95@gmail.com (A.Y.S.); 2 Laboratório de Fármaco-Toxicologia-UBIMedical, Universidade da Beira Interior, 6200-000 Covilhã, Portugal; 3 AlphaBiolabs, 14 Webster Court, Carina Park, Warrington WA5 8WD, UK; mbarroso@alphabiolabs.com; 4 Serviço de Química e Toxicologia Forenses, Instituto Nacional de Medicina Legal e Ciências Forenses—Delegação do Sul, 1169-201 Lisboa, Portugal; 5 Centro Académico Clínico das Beiras (CACB)-Grupo de Problemas Relacionados com Toxicofilias, 6200-000 Covilhã, Portugal

**Keywords:** antiepileptic drugs, monitoring, blood and derivates, urine, saliva, hair, exhaled air

## Abstract

Epilepsy remains a disease that affects many people around the world. With the development of new drugs to treat this condition, the importance of therapeutic drug monitoring continues to rise and remains a challenge for the medical community. This review article explores recent advances in the detection of antiepileptic drugs across various sample types commonly used for drug monitoring, with a focus on their applications and impact. Some of these new methods have proven to be simpler, greener, and faster, making them easier to apply in the context of therapeutic drug monitoring. Additionally, besides the classic use of blood and its derivatives, there has been significant research into the application of alternative matrices due to their ease of sample collection and capacity to reflect drug behavior in blood. These advances have contributed to increasing the efficacy of therapeutic drug monitoring while enhancing its accessibility to the population.

## 1. Introduction

Epilepsy is one of the most prevalent neurological diseases worldwide, affecting approximately 50 million individuals across all age groups. Besides neurological and cognitive complications, epilepsy also imposes significant psychological and social burdens due to stigma and discrimination [1]. Epilepsy is defined by the spontaneous recurrence of unprovoked seizures, meaning seizures not induced by transient systemic, metabolic, or toxic disorders. It can be classified as generalized, focal, unknown, and combined generalized and focal epilepsy. Generalized epilepsy is further divided into motor onset seizures, such as tonic and clonic, and non-motor onset seizures, such as myoclonic absence [1,2].

Factors such as the global increase in life expectancy and the rising proportion of individuals surviving events that often lead to epilepsy, such as birth trauma, traumatic brain injury, brain infections, and stroke, are expected to contribute to a higher prevalence of this condition worldwide. Thus, it is crucial to be attentive to the first symptoms [1]. A clinical diagnosis of epilepsy is made if there have been at least two unprovoked seizures occurring more than 24 h apart, or one unprovoked seizure with a recurrence probability of more than 60% over the subsequent 10 years [1,3].

The mainstay treatment strategy for seizures is medication management. However, much like the prescription of any other pharmaceutical agent, a clinician must balance efficacy with adverse events, and consider cost, drug interactions, patient preference, and availability [4]. Up to 70% of individuals with epilepsy could achieve seizure freedom with appropriate diagnosis and the use of cost-effective, commonly available antiseizure medicines, which can ultimately enable people with epilepsy to continue or return to a full and productive life [1,4].

While there is a multitude of different antiepileptic agents used in clinical practice today, they primarily act by interfering with one or more cellular mechanisms believed to cause seizures [5,6].

Antiepileptic drugs (AEDs) are categorized into two types: broad spectrum and narrow spectrum. Broad-spectrum AEDs treat a wide variety of seizure types and are a good initial choice, especially when the classification of seizure type is uncertain. These AEDs include, but are not limited to, levetiracetam, lamotrigine, zonisamide, topiramate, valproic acid, clonazepam, perampanel, clobazam, and rufinamide. Narrow-spectrum AEDs are primarily used for the treatment of focal or partial seizures. These include, but are not limited to, lacosamide, pregabalin, gabapentin, carbamazepine, oxcarbazepine, ezogabine, phenytoin, and vigabatrin [4].

Monotherapy is the ideal pathway for the treatment of seizures, but newer AEDs have had difficulty obtaining Food and Drug Administration approval as monotherapy agents due to the stringent requirements for approval. However, both the anecdotal evidence and current research suggest that second-generation AEDs appear to be an appropriate choice, as they have demonstrated similar efficacy compared to older AEDs and may be better tolerated [4].

There are several ways to classify AEDs, for example, Table 1 describes some AEDs according to their mechanisms of action. Some AEDs act on sodium channels by either blocking their repetitive activation (e.g., phenytoin and carbamazepine) or by enhancing their slow inactivation (e.g., lacosamide). Others target calcium channels by blocking T-type calcium channels (e.g., ethosuximide and valproic acid) or the N- and L-type calcium channels (e.g., zonisamide). Lamotrigine functions by blocking sodium channels, blocking N- and L-type calcium channels, and modulating the H-current. Topiramate acts by blocking sodium channels, alpha-amino-3-hydroxy-5-methyl-4-isoxazole propionic acid (AMPA) receptors, and inhibiting carbonic anhydrase. Other mechanisms of AED action include enhancing gamma-aminobutyric acid (GABA)-A receptors (e.g., phenobarbital and benzodiazepines), blocking *N*-methyl-*D*-aspartic acid (NMDA) receptors (e.g., felbamate), and opening neuronal potassium channels (e.g., ezogabine) [4].

AEDs can also be classified by their therapeutic usage. For instance:

- Simple partial seizures (carbamazepine, phenytoin, phenobarbital, primidone, valproate, gabapentin, and lamotrigine).
- Complex partial seizures (carbamazepine, phenobarbital, phenytoin, primidone, valproate, gabapentin, and lamotrigine).
- Partial with secondary generalized tonic–clonic seizures (carbamazepine, phenobarbital, phenytoin, primidone, valproate, gabapentin, and lamotrigine).
- Generalized absence seizures (clonazepam, ethosuximide, and valproate).
- Generalized myoclonic seizures (valproate).
- Tonic–clonic seizures (carbamazepine, phenobarbital, phenytoin, primidone, and valproate) [26].

Additionally, AEDs can be classified based on their chemical properties, such as barbiturates (e.g., phenobarbitone, mephobarbitone, and primidone), hydantoins (e.g., phenytoin and mephenytoin), iminostilbenes (e.g., carbamazepine), oxazolidinediones (e.g., trimethadione (troxidone)), succinimides (e.g., ethosuximide), aliphatic carboxylic acids (e.g., valproic acid), benzodiazepines (e.g., clonazepam and diazepam), acetylureas (e.g., phenacemide), newer drugs (e.g., progabide, vigabatrin, gabapentin, lamotrigine, felbamate, topiramate, and tiagabine), and miscellaneous (e.g., acetazolamide and dexamphetamine) [26].

Currently, approximately 30% of patients do not achieve satisfactory seizure control [5]. Additionally, many patients suffer from significant treatment-related adverse reactions, making therapeutic drug monitoring essential in epilepsy treatment to maximize clinical efficacy and minimize adverse drug reactions [2]. Drug monitoring is defined as the measurement and clinical use of drug concentrations in serum/plasma (or saliva) to adjust individual patient dosages, thereby tailoring it to each patient’s individual therapeutic requirements. It is most commonly applied to medications with a narrow therapeutic range; in this situation, we are referring to therapeutic drug monitoring (TDM). This monitoring has been used over the last 50 years to manage pharmacological variability within and between patients, during which time many drugs have been developed, enabling continuous advancements in this field and its impact on clinical practice [2,5,27].

TDM is crucial for all drugs where the serum concentration is expected to reflect the concentration and pharmacological action at the brain’s target site. The only exception is vigabatrin, an irreversible inhibitor of the enzyme responsible for GABA degradation and GABA transaminase. Vigabatrin can produce a prolonged effect on the brain, even when its serum concentration is declining or zero. Pharmacokinetic interactions may also be controlled through the use of TDM as changes in serum concentrations reflect alterations in metabolism [27].

In recent years, several new AEDs have been introduced to the market, with 27 AEDs now available internationally. The selection of the appropriate AED for different seizure types is of paramount importance, as some AEDs are specifically effective in certain seizure types. However, the efficacy and safety of these treatments rely heavily not only on the selection of the appropriate drug, but also on careful clinical monitoring throughout the course of therapy. TDM is a valuable tool for optimizing and individualizing AED treatment, allowing clinicians to adjust doses to achieve optimal therapeutic levels while minimizing the risk of toxicity or subtherapeutic dosing. Given the narrow therapeutic index of many AEDs, small changes in blood concentration can result in significant clinical consequences, including increased seizure frequency or adverse effects. Monitoring AED levels in the clinic provides real-time information on drug absorption, metabolism, and elimination, which can be influenced by factors, such as patient age, organ function, drug–drug interactions, and genetic variations [27]. Regular clinical monitoring is essential for ensuring that patients maintain therapeutic levels of AEDs, especially in cases where there are changes in the patient’s condition, the introduction of other medications, or alterations in adherence to treatment. Furthermore, TDM plays a critical role in long-term epilepsy management, helping to reduce the risk of treatment failure and minimizing potential side effects. The ability to individualize AED therapy through TDM not only improves seizure control, but also enhances patient safety and quality of life, making it a cornerstone of epilepsy treatment in clinical practice [28,29].

## 2. Strategies to Determine AEDs in Biological Specimens

As research progresses, the field of analytical techniques is constantly evolving, enabling the detection of drugs and their metabolites at extremely low concentrations. To achieve high performance metrics for any test, effective sample preparation before the detection step is crucial [30].

The three primary purification methods used for the extraction and concentration of analytes from biological samples are solid-phase extraction (SPE), liquid–liquid extraction (LLE), and protein precipitation (PP). Although these techniques are still in use today. They have several drawbacks. They require large sample volumes, emulsion formations in some cases, and the use of organic solvents, which generate significant waste. Additionally, they involve considerable manual work, making them less attractive [30,31].

Contemporary preconcentration techniques are generally divided into two major categories: liquid phase and solid-phase microextractions [32]. Solid-phase microextraction (SPME) is notably fast, widely applicable across various research domains, and demonstrates excellent sample purification outcomes. It is regarded as an extremely efficient technique for sample pretreatment that can be seamlessly integrated with separation or detection equipment. In-tube SPME (IT-SPME) is another advanced microextraction technique. This simple SPME format retains the benefits of traditional methods (solvent-free, fast, and cost-effective) and introduces a new “online extraction” mode that can be used with various mass spectrometry (MS) systems [32,33].

**Figure 1:**
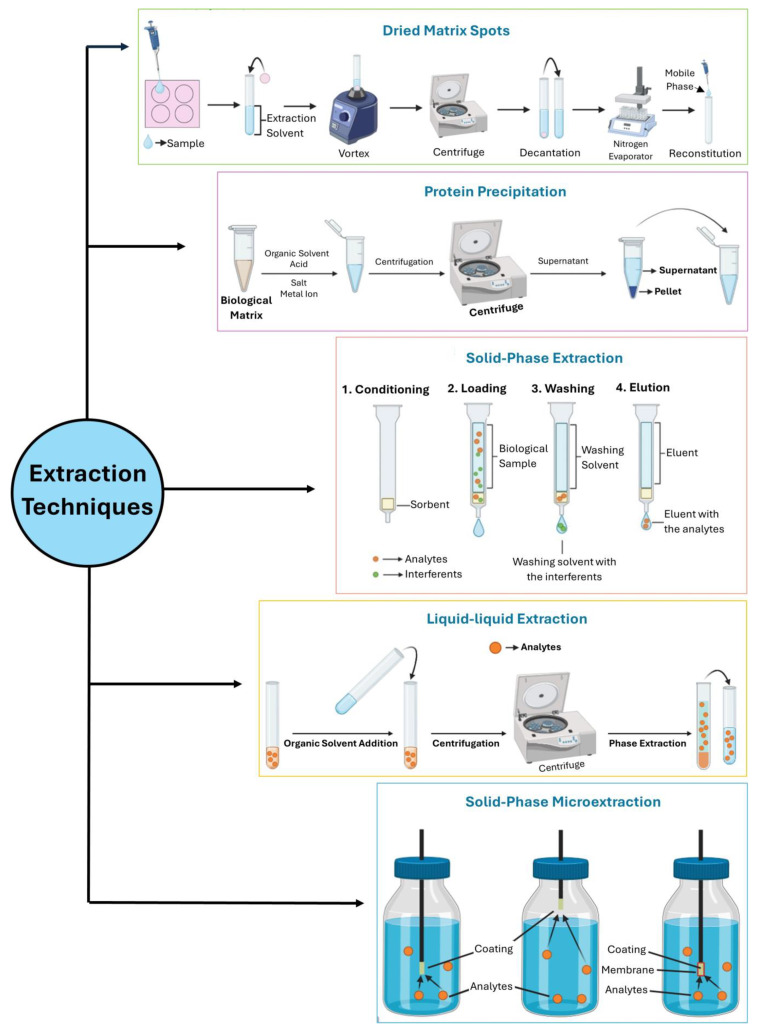
The five main extraction procedures that have been recently used.

An alternative method gaining popularity is dried blood spot (DBS) analysis, a technique developed based on the recent advancements in analytical sensitivity and technology [27]. Initially used for screening newborn metabolic abnormalities over the past 50 years, DBS analysis has recently become increasingly relevant for determining both small and large compounds, particularly in clinical practice, toxicological and pharmacokinetic investigations, and sports drug testing. This technique offers several advantages for therapeutic drug monitoring in future clinical practice due to its reduced invasiveness, potential for automation, lower risk of infection, cost-effectiveness, streamlined sampling, storage, and transportation, and the ability to enhance the stability of many analytes [34]. Building on the advantages of DBS sampling, an adaptation to oral fluids has been developed and termed dried saliva spots (DSSs). This technique has proven to be an excellent alternative to neat oral fluids for pharmacokinetic evaluations of drugs [35]. By facilitating sample collection, DSS sampling reduces the risk of sample substitution or adulteration, owing to the possibility of supervision during collection. This method is also particularly valuable for monitoring AEDs, offering new solutions for handling, sampling, storage, and transportation due to its non-invasive collection method [27,34,35]. In Figure 1, the previously described extraction procedures are shown.

The absence of derivatization steps and the high specificity and sensitivity of liquid chromatography tandem mass spectrometry (LC-MS/MS) and liquid chromatography with mass spectrometry (LC-MS) make these methods highly relevant for detection. Additionally, these methods can handle complex matrices with ease. Their high sensitivity compensates for the low volume of alternative samples typically available. However, LC-MS still faces challenges, such as ion suppression or enhancement, when dealing with complex matrices [36].

As mentioned earlier, the number of available AEDs on the market has increased, highlighting the importance of selecting the appropriate medication for specific seizure types. Concurrently, the development and validation of new methods have expanded to address the need for effective control in maximizing clinical efficacy and minimizing adverse drug reactions. Given that therapeutic drug monitoring is crucial for drugs where plasma concentrations reflect the drug’s concentration and pharmacological action at the target site, researchers have been developing more methods for determining AEDs in serum, plasma, and whole blood. These new methods aim to address the limitations of previous approaches while adapting alternative matrices that offer less invasive collection methods and can reflect drug concentrations in the blood [27].

Based on this information, the present work provides a comprehensive review of the various applications and methods for determining AEDs in biological samples. For clarity, this section is organized by the type of biological sample in which AEDs can be detected. For each type of biological sample, the main developments in the determination of these compounds are discussed.

A search was conducted in both PubMed and ISI Web of Science databases using the following combinations of keywords: “determination of antiepileptic drugs” with the biological samples “blood”, “urine”, “oral fluid”, “hair”, “exhaled air”, “breast milk”, “alternative specimens”, and "other samples”, restricting the search to the last three years (from 2019 onward).

### 2.1. Blood and Derivates

Blood is considered a traditional matrix due to its historical use for drug testing in clinical and forensic toxicology, being one of the matrices in which higher drug levels can be detected [37]. The quantitative analysis of whole blood and blood-derived matrices is preferred due to their ability to correlate drug concentration with potential pharmacological effects. These matrices can also indicate recent drug use (hours) and are less prone to adulteration. Despite these advantages, blood and its derivatives still present several drawbacks, such as the invasive nature of collection, which requires qualified personnel, a short detection window, and the need for matrix extraction [37,38]. Table 2 summarizes the analytical procedures developed and published for the determination of AEDs in blood and its derivatives from 2020 to the present year.

Overall, plasma is the most commonly used sample for these methods, representing over 60% of the published analytical developments for the determination of AEDs since 2020. This preference stems from plasma’s ability to effectively correlate drug concentrations with their potential pharmacological effects, making it a reliable choice for therapeutic drug monitoring, where therapeutic and toxic levels are well-documented. Traditional sample preparation techniques, particularly PP, remain the most widely applied for blood and its derivatives, accounting for more than 50% of the methods developed. LLE follows with approximately 15% of published methods. Despite their advantages, these classic procedures are typically applied to smaller sample volumes (<100 µL), aligning with the trend toward miniaturization in new methodological approaches. This trend is facilitated by the increasing sensitivity of modern analytical equipment, as reflected by the substantial use of LC-MS/MS methods, which account for around 56% of developments for AEDs in blood and its derivatives. Mass spectrometry (MS) is the chosen detector in 78% of publications since 2020. The enhanced sensitivity and specificity of these advanced instruments often allow for lower sample volumes and simpler sample preparation techniques, explaining the continued popularity of PP, especially when paired with LC-MS or LC-MS/MS. However, for gas chromatography-mass spectrometry (GC-MS) analyses, LLE is preferred.

Valproic acid is the most frequently detected AED in blood and its derivatives (see Table 2). Gu et al. [42] developed a method for detecting the free fraction of valproic acid in human plasma that is applicable across various medical facilities and requires only 200 µL of plasma. By combining centrifugal ultrafiltration and PP with dichloromethane, they achieved a limit of quantification (LOQ) of 0.56 µg/mL, demonstrating good specificity, stability, and cost-effectiveness. An interesting article was published by Schaefer et al. [54]. In this work, an LOQ of 10 µg/mL was achieved using 50 µL of serum samples and bioanalytical solid-phase microextraction (BioSPME). BioSPME, a new microextraction strategy, involves serum acidification, the direct immersion of BioSPME tips, agitation, and methanol desorption. This method provided comparable results to an LLE-based GC-MS method, highlighting its potential as an alternative sample preparation technique. In recent years, volumetric absorptive microsampling (VAMS) and dried blood spot (DBS) sampling have emerged as valuable techniques for sample collection and analysis, thanks to their numerous advantages. Both methods offer a minimally invasive approach to blood sampling, which greatly reduces patient discomfort and the risks associated with traditional venipuncture. VAMS employs a specialized device to absorb a precise volume of blood [103], while DBS involves depositing blood onto filter paper [104]. These techniques simplify the sample collection process, making them suitable for use in various settings, including remote locations. One of the significant benefits of VAMS and DBS methods is the stability of the samples. Both types can be stored at room temperature, which streamlines transportation and long-term storage, eliminating the need for cold chain logistics [105]. This stability is crucial for accurate analysis over time. Additionally, the cost-effectiveness of these methods is evident in the reduced need for extensive laboratory equipment and complex sample preparation procedures, leading to lower costs for both sample collection and analysis. The reduced risk of infection, owing to the less invasive nature of these techniques, further adds to their appeal. The applications of VAMS and DBS methods have expanded significantly [106]. In clinical settings, these methods are increasingly employed for monitoring drug levels, including AEDs, due to their convenience and effectiveness, and they facilitate routine therapeutic drug monitoring with minimal patient discomfort. An example of this application is the work of Velghe et al. [41]. The authors obtained an LOQ of 25 µg/mL using both DBS and VAMS techniques with LC-MS/MS. They also detected carbamazepine with an LOQ of 2 µg/mL, and carbamazepine-10,11-epoxide and phenobarbital with an LOQ of 1 µg/mL. Although both methods showed similar LOQs, the VAMS technique demonstrated better recovery rates. Li et al. [50] presented a robust method for quantifying valproic acid using LC-MS/MS and dried plasma spots (DPSs), achieving an LOQ of 10 µg/mL with just 40 µL of plasma. The simplicity, flexibility, and affordability of the PP method with acetonitrile provided high recovery rates [41,42,50,54,103,104,105,106].

Other applications using DBS and DPS methods are the studies of Möller et al. [34] and Rmandić et al. [61]. Möller et al. [34] developed an LC-MS/MS method to quantify five drug metabolites and 22 medications used by epilepsy patients with the DBS method. Their findings confirmed that DBS analysis is feasible, with all analytes detected in 20 μL of blood. However, conversion factors are needed to compare DBSs and serum concentrations accurately, and further investigation is required to clinically validate the results [34,61].

Rmandić et al. [61] developed a ultra-high-performance liquid chromatography tandem mass spectrometry (UHPLC–MS/MS) method for zonisamide determination in DPSs and DBSs. With a runtime under 2.5 min and volumes of 50 μL for DBSs and 30 μL for DPSs, they achieved LOQs of 0.125 µg/mL for DBSs and 0.250 µg/mL for DPSs. This method proved economical and environmentally friendly, facilitating sample preparation by directly dissolving zonisamide from DBS/DPS cards in the mobile phase, and was effective for both blood and plasma quantifications [61].

Lacosamide is another AED frequently detected in blood and its derivatives. Mouskeftara et al. [39] achieved an LOD of 0.1 µg/mL and an LOQ of 0.5 µg/mL using LLE and GC-MS. The application of alkaline LLE with NaOH of 0.01 M and ethyl acetate effectively extracted lacosamide from blood samples, allowing for the detection of concentrations ten-times lower than the therapeutic range. Zhao et al. [47] developed and validated an ultra-high-performance liquid chromatography coupled diode array detector (UHPLC-DAD) method for quantifying lamotrigine, oxcarbazepine, and lacosamide in serum, obtaining the same LOQ of 0.5 µg/mL as Mouskeftara et al. [39] using a PP technique. Both methods exhibited high recovery rates, with Mouskeftara et al. [39] achieving recoveries between 78.56% and 121.90%, and Zhao et al. [47] between 96.58% and 106.22%. Notably, Zhao et al.’s method required an approximately five-times-less sample volume than Mouskeftara et al.’s [39,47].

Regarding the use of microextraction techniques, there are several interesting applications. To determine oxcarbazepine concentrations in human plasma and urine, Erarpat et al. [32] developed a sensitive, rapid, and environmentally friendly method involving vortex-assisted switchable hydrophilicity solvent-based liquid phase microextraction (VA–SHS–LPME) before GC-MS analysis. The switchable hydrophilicity solvents’ miscibility, altered by carbon dioxide, enabled efficient analyte extraction without the need for disperser solvents. The VA-SHS-LPME method achieved an LOD of 6.2 μg/kg and an LOQ of 21 μg/kg with 1 g of plasma. Hu et al. [33] coupled in-tube solid-phase microextraction (IT-SPME) directly with mass spectrometry (MS) using an open tubular column coated with a polymer for quantifying carbamazepine and oxcarbazepine in plasma and urine samples. This new “online extraction” mode allows IT-SPME to integrate seamlessly with various MS equipment, offering a high extraction efficiency and low LODs and LOQs [32,33].

As previously indicated, the most commonly used instrumental techniques are GC and LC coupled with MS and/or MS/MS. However, there are simpler methods that utilize more economical detection systems. One such study is described by Seyfinejad et al. [45]. The authors developed a method for determining the free fraction of phenytoin in plasma using electromembrane extraction (EME) and capillary electrophoresis coupled with diode array detection (CE-DAD). EME, based on extracting charged compounds using an electric field, achieved an LOD of 0.005 μg/mL and an LOQ of 0.03 μg/mL [45].

### 2.2. Urine

Like blood, urine is also considered a traditional matrix. It is the preferred matrix for systematic toxicological analysis because metabolites are usually present and concentrations are comparatively high. Metabolite detection can reduce the risk of a false negative and aid in the substance identification process. Additionally, urine has relatively extended detection windows, making it particularly suitable for workplace drug testing and forensic investigations, such as drug-facilitated crimes [38]. Although urine collection is less invasive and potentially more attractive for medication monitoring, it lacks established clinical ranges, which results in it not being applied often in the TDM process. This makes it exceedingly challenging to create new techniques that reveal appropriate concentration ranges, resulting in a decreased number of publications on this matter [107]. Moreover, urine samples can be easily adulterated [38,107].

While researching the literature for articles on the determination of antiepileptic drugs in urine from 2020 to the present year, only five different articles were found (Table 3). GC-MS instrumentation was mainly used when a single AED was to be quantified. In contrast, the LC-MS/MS system, due to its high sensitivity, was applied to a multi-method development for nine target compounds.

As mentioned before, Erarpat et al. [32] developed a new, sensitive, rapid, and environmentally friendly analytical method that included VA–SHS–LPME prior to GC-MS analysis. With urine, the authors achieved an LOD of 6.2 µg/kg, an LOQ of 21 µg/kg, and a recovery rate ranging from 97% to 100%. These results were particularly impressive as this was the first application of VA-SHS-LPME for determining oxcarbazepine in the literature [32].

Feng et al. [107] developed a new, fast, LC-MS/MS method capable of determining eslicarbazepine, carbamazepine-10,11-epoxide, topiramate, phenytoin, oxcarbazepine, carbamazepine, levetiracetam, lamotrigine, and 4-hydroxyphenytoin simultaneously in under four minutes of runtime. By combining SPE for sample preparation and LC-MS/MS, the authors achieved low LODs and LOQs for all nine AEDs and good recoveries for most of them, using only 25 µL of urine. In comparison to Erarpat et al.’s green method, Feng et al. [107] managed to achieve lower LODs and LOQs for oxcarbazepine determination in urine with a shorter runtime [107].

The method developed by Hu et al. [33] for detecting carbamazepine and oxcarbazepine in plasma is also an excellent tool for analyzing urine samples. The LODs of 0.08 ng/mL and 0.10 ng/mL, and LOQs of 0.30 ng/mL for both carbamazepine and oxcarbazepine in urine, are significantly lower than those obtained using previous methods [33].

Regarding valproic acid, Namera et al. [64] developed a simple and cost-effective method using LLE and GC-MS, achieving an LOD of 1 µg/mL and recoveries ranging from 86.2% to 98% with 100 µL of urine [64].

Lastly, Mariyappan et al. [108] developed an electrochemical sensor for the determination of carbamazepine in urine. By functionalizing a glassy carbon electrode with produced carbon nanofiber, they created an electrochemical sensor based on a functionalized gadolinium vanate nanostructure. Using this electrochemical sensor, they achieved an LOD of 0.0018 µM and an LOQ of 0.006 µM [108].

### 2.3. Oral Fluid

Oral fluid’s safe (or more usually named saliva), straightforward, and non-invasive collection process has contributed to the recent increase in interest in its toxicological analysis applications. This specimen can be considered simpler than traditional matrices and might present fewer interferents, subsequently resulting in more accurate drug analysis. Oral fluid can be a useful tool for drug monitoring, as its drug levels are assumed to represent the concentration of free drug in plasma. It also reflects recent drug use (hours) and does not require privacy during sample collection, reducing the chances of adulteration. Nonetheless, oral fluid can be easily contaminated during collection, and only a small volume of sample can be obtained at a time. Additionally, the collection method can influence oral fluid’s pH and drug concentrations [38,111,112].

Interest in alternative specimens, such as oral fluid, for drug determination has been growing. Studies have shown a strong correlation between saliva and the plasma levels of certain AEDs, making saliva a reliable alternative for drug monitoring [111,113]. While the interest is growing, the number of comprehensive studies and standardized protocols for saliva monitoring of AEDs remains limited. In fact, only five articles related to the determination of AEDs in oral fluid were found from 2020 to the present year (Table 4).

Kruizinga et al. [57] developed an LC-MS method to determine clonazepam in 20 µL of oral fluid to investigate the correlation between clonazepam levels in this sample and plasma. To forecast plasma concentrations from oral fluid samples, the authors evaluated a population pharmacokinetics model to explain this correlation. This novel approach proved to be effective [57].

In a different approach, Tommasini et al. [114] utilized surface-enhanced Raman scattering (SERS) to detect perampanel in saliva samples. SERS can identify low drug concentrations with analytical capabilities comparable to HPLC. Thus, the study explored the use of SERS for the therapeutic drug monitoring of perampanel for the first time. This new method required only 10 µL of oral fluid [114].

Similar to the previous studies, the next four articles focus on developing or optimizing methods for the drug monitoring of various antiepileptic drugs. Carona et al. [115] developed an HPLC-DAD method for monitoring levetiracetam, *S*-licarbazepine, carbamazepine, lacosamide, and carbamazepine-10,11-epoxide, achieving satisfactory recovery ranges for all five AEDs. This method achieved statistically significant correlations between the analyzed AEDs [115].

Kim et al. [51] utilized an LC-MS/MS system to detect perampanel in human saliva samples, determining both the total and free concentrations of perampanel with only 50 µL and 1 mL of oral fluid, respectively. This study demonstrated that oral fluid could be used for the drug monitoring of perampanel, as the total concentration of perampanel in oral fluid showed a linear correlation with the free concentration in plasma [32].

Zhang et al. [66] developed a straightforward, sensitive, and reliable UHPLC-MS/MS method to determine levetiracetam concentrations in oral fluid samples from pregnant Chinese women with epilepsy. This method achieved an LOQ of 1 ng/mL with a simple sample treatment (PP) and recoveries ranging from 108.8% to 113.5% [48].

Finally, Franco et al. [52] used a validated HPLC-UV method to compare rufinamide concentrations in saliva and plasma samples to assess the viability of saliva as an alternative for the TDM of rufinamide. The authors achieved excellent LOQ and LOD values, 0.25 µg/mL and 0.05 µg/mL, respectively, confirming the applicability of saliva for the TDM of rufinamide, despite the lower concentrations in saliva compared to plasma samples [33].

### 2.4. Hair

Hair samples can play a significant role in drug monitoring due to their unique properties. Unlike blood and urine, hair provides a long-term record of drug exposure, as substances incorporated into hair are retained for months or even years, depending on hair growth and length. This makes hair analysis particularly useful for assessing chronic drug use or long-term compliance with a prescribed regimen. One of the key advantages of using hair samples for drug monitoring is their stability. Hair is less likely to degrade or be contaminated compared to other biological matrices, making it a reliable source for historical drug exposure data. Additionally, hair samples can be collected non-invasively and without the need for specialist medical staff, which can simplify the monitoring process. However, this specimen is prone to environmental contamination, particularly from cosmetic products, which can affect the results. Furthermore, the interpretation of hair drug concentrations can be complex, as they may not directly correlate with current drug levels or effects. This can make it challenging to assess recent drug use accurately [117,118].

The study by Kim et al. [119] is the only article found from 2020 to the present that presents a method for determining AEDs in hair samples. These authors developed a novel, rapid, and efficient analytical method based on LC-MS/MS that allows for the simultaneous detection of topiramate, phenytoin, and six barbiturates in hair samples. For sample preparation, they explored three different conditions: methanolic extraction, LLE after basic digestion, and methanolic extraction with 1% hydrochloric acid. In the methanolic extraction process, hair samples are evaporated to dryness at 45 °C in glass tubes under a mild nitrogen stream after being incubated with 2 mL of methanol (for simple methanolic extraction) or acidified methanol (for acidified methanolic extraction) at 38 °C for 16 h. The residues are then treated in a glass insert micro-vial with a 0.22 µm hydrophilic syringe filter after reconstitution in 100 µL of mobile phase. For the LLE procedure, the hair samples undergo hydrolysis with 1 mL of 1 M NaOH at 90 °C for 30 min. Following this, 200 µL of acetic acid and 300 µL of 0.1 M sodium acetate buffer (pH 4.5) are added to acidify and digest the hair samples before extraction with 2 mL of n-hexane/ethyl acetate (1/1, *v*/*v*) for 5 min. The residues are then reconstituted in 100 µL of mobile phase prior to injection. The LOD and LOQ were 0.01 and 0.02 ng/mg for topiramate and in the ranges of 0.25–0.5 and 0.5–1 ng/mg for the other drugs, respectively. This method was applied to authentic hair samples from two drug users. In a segmental analysis of one female subject, phenobarbital concentrations ranged from 0.2 to 17.1 ng/mg. In another female subject, topiramate concentrations ranged from 0.19 to 0.93 ng/mg [119].

Certainly, the use of hair for the determination of AEDs is more prevalent in forensic toxicology than in clinical toxicology, due to the characteristics of this sample mentioned earlier.

### 2.5. Exhaled Air

Breath analysis has recently emerged as a valuable diagnostic tool, on par with blood and urine, for a broad range of analytical applications. These include monitoring biological responses, assessing health conditions, evaluating metabolic kinetics, studying toxicological effects, and chemical exposures, as well as conducting multiple time-point assessments [120]. The ease of sample collection, the ability for continuous sampling, and the lack of need for sample preparation are key reasons why exhaled air has long been used to monitor blood alcohol levels and enhance road safety. Given these advantages, breath analysis is now being explored as a tool for drug monitoring, particularly in pediatric cases [120,121,122].

The study by Awchi et al. [55] exemplifies this application and is notably the only article found on the detection of AEDs in alternative matrices from 2020 to the present. This study demonstrated the potential of real-time breath analysis for predicting valproic acid concentrations in clinical settings. It introduced a hybrid method combining offline breath specimen collection with secondary electrospray ionization coupled with high-resolution mass spectrometry (SESI-HRMS) for real-time analysis [55].

While real-time analysis eliminates sample manipulation, thus preserving the biochemical integrity of the sample, it is not feasible for screening large populations due to the difficulty some patients may have in performing controlled exhalations. To address this issue, the authors employed a custom-made nalophan bag, with a capacity of approximately 2 liters, to collect samples away from the mass spectrometer. This approach facilitated the transport and subsequent analysis of the stability of exhaled compounds associated with valproic acid by SESI-HRMS. The results showed that this hybrid method could detect around 55% of approximately 1200 mass spectral traits commonly found in breath, and its performance was comparable to real-time analysis. Additionally, the method demonstrated a stable signal intensity over four years of data collection, highlighting its potential for long-term patient monitoring [55].

It is worth noting that the authors compared three different analytical methods with varying quantitative capabilities in terms of sensitivity, accuracy, and precision. These included the enzyme-multiplied immunoassay technique (EMIT) for total valproic acid quantification in serum, versus offline and GC-MS for free valproic acid in serum, versus offline methods. This comparison underscores the importance of carefully evaluating the prediction capabilities of these methods, especially when interpreting results outside the therapeutic range [55].

Overall, this new technique shows significant promise for clinical applications, particularly when personalizing treatment for patients with epilepsy. The authors believe that this approach could enable widespread screening using a CE-marked in vitro diagnostic breath test, beneficial for a diverse patient population, including children and individuals with intellectual disabilities [55].

Another interesting study is the one published by Seyfinejad et al. [123]. The authors analyzed phenytoin in exhaled breath condensate using electromembrane extraction, a selective technique for isolating ionized molecules from samples. This method utilized a supported liquid membrane impregnated with 1-octanol, through which phenytoin was extracted from the exhaled breath condensate into an alkaline aqueous solution under a controlled electric field. The extraction process achieved optimal results with a 102-fold enrichment factor at 15 V over a 15 min period, with stirring at 750 rpm and a donor pH of 11.

Following extraction, the samples were analyzed using CE. The results demonstrate that this method is highly selective and precise, with no interfering peaks detected. The limit of detection for phenytoin was 0.001 µg/mL, indicating excellent sensitivity. The intra- and inter-day precision values were reported to be below 14%.

This method was successfully applied to real samples from patients receiving phenytoin therapy, proving it to be an effective, non-invasive alternative to blood sampling for TDM. Furthermore, the study concluded that electromembrane extraction combined with CE is a robust, low-cost, and highly sensitive approach, with potential for broader applications in clinical settings.

The researchers suggest that this method may offer a more convenient way to monitor AED levels in patients, supporting more frequent and less invasive monitoring.

### 2.6. Breast Milk

Breast milk serves as a vital biological sample in toxicology, especially when studying drug transfer and exposure in neonates. Its unique composition, including nutrients, hormones, and immune factors, makes it the optimal nutrition source for infants. However, it is also a route through which infants can be exposed to medications and environmental toxins ingested or absorbed by the mother. Analyzing breast milk is critical in evaluating the safety of maternal drug use during breastfeeding and assessing the potential risks of toxic substance exposure to the infant. Given its non-invasive collection process and the direct link between maternal and infant exposure, breast milk is an important medium for monitoring drug excretion and informing clinical decisions on safe breastfeeding practices for mothers on medication [124]. Dinavitser et al. [125] published a study concerning the use of this sample in the monitoring of AEDs. The study investigates the excretion of levetiracetam, an antiepileptic drug, into human breast milk and its impact on breastfed infants. Twenty women with epilepsy, treated with levetiracetam, participated in the study, and breast milk and serum samples were collected to measure the drug’s concentrations. The results indicate that the breast milk/serum ratio of levetiracetam is close to 1, suggesting that the drug passes efficiently into breast milk. The relative infant dose was above the safety threshold of 10% in fully breastfed infants, with three cases of somnolence reported in infants, which resolved after switching to partial breastfeeding. These findings suggest a need for the careful monitoring of breastfeeding infants exposed to levetiracetam, considering potential short- and long-term safety risks.

No studies have been found, following the previously established search criteria, on the determination of antiepileptic drugs in other types of samples.

## 3. Conclusions

To conclude, this review highlights several emerging areas and methods that have shown promising results in detecting AEDs in biological samples. Over the past four years, researchers have increasingly focused on utilizing alternative samples to blood/plasma, primarily due to the advantages of less invasive collection procedures. Additionally, efforts have been made to optimize existing methods and develop new ones to reduce analysis time and simplify procedures, with a focus on making them more environmentally friendly.

As anticipated, blood and derivatives remain the most extensively studied matrices, while oral fluid and urine show positive results as viable alternatives. Both matrices have shown promise due to their ease of collection, and innovative approaches have even led to the development of an electrochemical sensor for detecting carbamazepine in urine with high precision and efficiency. Unfortunately, despite its potential for monitoring long-term drug exposure, hair has not seen significant advancements in the detection of AEDs. It is surprising that only one relevant study has been identified from 2020 to the present year, given the advantages hair offers. Similarly, exhaled air remains an underexplored matrix, though it has shown potential in a method capable of predicting the free fraction of valproic acid.

Overall, the evolution of methodologies for detecting AEDs in biological samples has unveiled numerous new avenues for improving therapeutic drug monitoring. Continued advancements in traditional sample analysis are expected, and the exploration of alternative matrices promises to yield a variety of new methods, given the potential demonstrated in the limited number of studies to date.

## Figures and Tables

**Table 1:** Classification of some AEDs according to their mechanism of action.

Mechanism of Action	Drugs	Reference
Modulation of voltage-gated sodium channels	**Carbamazepine:** Inhibition of voltage-gated sodium channels; **Oxcarbamazepine:** Inhibition of voltage-gated sodium channels; **Eslicarbazepine:** Blockade of voltage-gated sodium channels; **Fosphenytoin:** Inhibition of voltage-gated sodium channels; **Lamotrigine:** Inhibition of voltage-gated sodium channels.	[4,7]
GABA receptors modulation	**Vigabatrin:** Inhibition of GABA transaminase; **Clobazam:** Allosteric modulation of GABA-A receptors; **Clonazepam:** Allosteric modulation of GABA-A receptors; **Primidone:** Binding to the GABA-A receptor, prolonging its open state to allow for more chloride influx and consequent cellular hyperpolarization; **Tiagabine:** Potent, selective, and competitive inhibition of the GAT-1 GABA transporter, blocking both neuronal and glial GABA re-uptake.	[4,7,8,9,10]
Modulation of calcium channels	**Ethosuximide:** Blockade of T-type calcium channels in thalamocortical neurons; **Gabapentin:** Binding with high affinity to α2δ-1 subunits of the voltage-gated calcium channel, causing conformational changes; possible action on GABA disposition; **Pregabalin:** Binding with high affinity to α2δ-1 subunits of the voltage-gated calcium channel, causing conformational changes; **Trimethadione:** Reduction in T-type calcium currents in thalamic neurons, including thalamic relay neurons via the inhibition of voltage dependent T-type calcium channels.	[7,11,12,13]
Carbonic anhydrase modulation	**Acetazolamide:** Inhibition of carbonic anhydrase.	[7,14]
Modulation of glutamate receptors and others	**Perampanel:** Non-competitive blockade of AMPA receptors.	[7,15]
Unknown mechanism of action	**Levetiracetam:** Possibly effective due to the binding of SV2A; **Cannabidiol:** Mechanism still unknown.	[4,7]
Several mechanisms of action	**Phenytoin:** Blockade of voltage-gated sodium channel; decreased synaptic transmission; smaller changes in ionic gradients involving the sodium–potassium ATPase pump; inhibition of calcium–calmodulin phosphorylation; **Lacosamide:** Stabilization of hyperexcitable membranes and inhibition of repetitive neural firing via the slow inactivation of voltage-gated sodium channels; binding to CRMP2; **Zonisamide:** Blockade of sodium channels; blockade of calcium channels; Inhibition of carbonic anhydrase; **Phenobarbital:** Binding to the GABA-A receptor, prolonging its open state to allow for more chloride influx and consequent cellular hyperpolarization; blockade of L- and N- type calcium currents; competitive blockade of AMPA receptors; **Valproate:** Augmentation of GABA concentrations; voltage-gated sodium channel inhibition; mild inhibition of T-type calcium currents; **Cenobamate:** Allosteric modulation of GABA-A receptors in hippocampal neurons, with effects on both phasic and tonic inhibitory currents and on recombinant synaptic and extra synaptic GABA-A receptor isoforms; inhibition of the persistent sodium current more potently than the transient sodium current; **Valproic Acid:** Enhancement of the expression of glutamic acid decarboxylase to promote the release of GABA from presynaptic terminals; prevention of the catabolism of GABA by inhibition of GABA transaminase; positive allosteric modulator at the GABA-A receptor; **Topiramate:** Blockage of carbonic anhydrase to a modest extent; blockade of voltage-gated sodium channels; GABA transmission enhancement; NMDA receptor antagonization; **Zonisamide:** Inhibition of carbonic anhydrase; blockade of sodium channels; blockade of calcium channels; **Felbamate:** Blockade of the NMDA subtype of glutamate receptor; blockade of sodium channels; effects on high-voltage-activated calcium channels; promotion of GABA responses at GABA-A receptors.	[4,7,16,17,18,19,20,21,22,23,24,25]

AMPA: α-amino-3-hydroxy-5-methyl-4-isoxazolepropionic acid; ATPase: adenosin triphosphatase; CRMP2: collapsin response mediator protein 2; GABA: gamma-aminobutyric acid; GABA-A: gamma-aminobutyric acid type A; GABAtransaminase: gamma-aminobutyric acid transaminase; GAT1: gamma-aminobutyric acid (GABA) transporter 1; NMDA: N-methyl-D-aspartate; SV2A: synaptic vesicle protein 2.

**Table 2:** Sample pretreatment and determination of AEDs in blood samples and derivatives.

Compounds	Matrix	Volume	Extraction Method	Detection Method	LOD	LOQ	Recovery (%)	Reference
Lacosamide	Blood	500 µL	LLE (methanol, NaOH 0.01 M, and ethyl acetate)	GC-MS	0.1 µg/mL	0.5 µg/mL	78.6–121.9	[39]
Valproic Acid	Plasma	300 μL	PP (85:15 (*v*/*v*) solution of perchloric acid–ethylene glycol and 50% ammonium acetate solution)	2D-LC-UV	1.00 μg/mL	n.s.	95.2–98.0	[40]
(a) Valproic Acid (b) CBZ (c) CBZ-E (d) Phenobarbital	Blood	n.s.	DBS	LC-MS/MS	n.s.	(a) 25 µg/mL (b) 2 µg/mL (c) n.s. (d) 1 µg/mL	(a) 58.7 ± 8.33 (b) 62.6 ± 9.36 (c) 61.0 ± 9.99 (d) 61.2 ± 9.79	[41]
(a) Valproic Acid (b) CBZ (c) CBZ-E (d) Phenobarbital	Blood	n.s.	VAMS (acetonitrile/water (80/20 *v*/*v*))	LC-MS/MS	n.s.	(a) 25 µg/mL (b) 2 µg/mL (c) n.s. (d) 1 µg/mL	(a) 85.2 ± 6.1 (b) 86.4 ± 5.9 (c) 91.4 ± 4.6 (d) 93.7 ± 4.6	[41]
Valproic Acid	Plasma	200 μL	CF-UF/PP (dichloromethane)	GC-FID	n.s.	0.56 µg/mL	101.45 ± 2.08 to 102.58 ± 3.38	[42]
Oxcarbazepine	Plasma	1.0 g	VA–SHS–LPME (N,N-dimethylbenzylamine, distilled water (1:1, *v*/*v*), dry ice, and sodium hydroxide)	GC-MS	6.2 μg/kg	21 μg/kg	97–100	[32]
Lacosamide	Plasma	n.s.	PP (methanol:water (50:50, *v*/*v*), acetonitrile:methanol, 50:50 (*v*/*v*), and 0.1% formic acid in water (80:20, *v*/*v*))	UHPLC-MS/MS	n.s.	n.s.	97.2–99.7	[43]
Levetiracetam	Plasma	300 μL	PP (methanol)	HCLC-UV-PDA	n.s.	6 µg/mL	n.s.	[44]
Phenytoin	Plasma	n.s.	EME	CE-DAD	0.005 μg/mL	0.03 μg/mL	n.s.	[45]
Sulthiame	Serum/Plasma	50 μL	PP (acetonitrile)	RP-HPLC–UV	0.19 μL/mL	0.58 μL/mL	≈100	[46]
(a) Lacosamide (b) Oxcarbazepine (c) Lamotrigine	Serum	90 μL	PP (protein precipitator)	UHPLC-DAD	(a) 0.25 μg/mL (b) 0.50 μg/mL (c) 0.25 μg/mL	(a) 0.5 µg/mL (b) 2.5 µg/mL (c) 0.5 µg/mL	96.6–106.2	[47]
(a) Perampanel (b) Lamotrigine	Plasma	200 μL	LLE (ethyl acetate)	HPLC-DAD	n.s.	(a) 0.03 µg/mL (b) 0.25 µg/mL	(a) 90.0–114.6 (b) 93.3–112.9	[48]
Pregabalin	Serum	(a) n.s. (b) 100 μL	(a) n.s. (b) n.s.	(a) IT (b) LC-MS/MS	n.s.	n.s.	n.s.	[49]
Valproic Acid	Plasma	(a) 40 μL (b) 30 μL	(a) DPS/PP (water–ACN, 50:50, *v*/*v*)) (b) PP (acetonitrile)	(a) LC–MS/MS (b) LC–MS/MS	n.s.	10 µg/mL	(a) 82.6–86.0 (b) 98.4–99.8	[50]
Perampanel	Plasma	(a) 50 µL (b) 1000 µL	n.s.	LC-MS/MS	n.s.	0.5 ng/mL	n.s.	[51]
Rufinamide	Plasma	250 µL	LLE (methanol, ammonium hydroxide solution pH 9.25, and dichloromethane)	HPLC-UV	0.05 μg/mL	0.25 μg/mL	94.1 ± 4.7	[52]
**(a) Brivaracetam** **(b) Carbamazepine** **(c) Carbamazepine-epoxide** **(g) Gabapentin** **(h) Lacosamide** **(i) Lamotrigine** **(j) Lamotrigine-13C,15N4** **(k) Levetiracetam** **(m) 10-OH-Carbazepine** **(n) Perampanel** **(o) Phenytoin** **(p) Pregabalin** **(q) Primidone** **(u) Rufinamide** **(w) Stiripentol** **(x) Sultiame** **(y) Topiramate** **(b’) Zonisamide**	Blood	20 μL	DBS	LC-MS/MS	n.s.	n.s.	n.s.	[34]
Rufinamide	Plasma	50 μL	PP (methanol)	RP-HPLC-UV	n.s.	0.5 µg/mL	n.s.	[53]
Valproic Acid	Serum	50 µL	BioSPME (HCl 0.1 M, methanol)	GC-MS	n.s.	10 µg/mL	n.s.	[54]
Gabapentin	Serum	20 μL	PP (methanol, 95:5(*v*/*v*) 10 mM ammonium formate:methanol, and 0.1% formic acid)	LC-MS/MS	n.s.	0.1 µg/mL	n.s.	[55]
Clonazepam	Plasma	n.s.	UA-EME	CE-DAD	3.0 ng/mL	0.01 µg/mL	58	[56]
Clonazepam	Plasma	50 μL	n.s.	LC-MS	n.s.	2 µg/mL	n.s.	[57]
(a) Fenfluramine (b) Norfenfluramine	Plasma	n.s.	PP	LC-MS/MS	n.s.	n.s.	n.s.	[58]
Carbamazepine	Plasma	5 µL	PP (methanol)	LC-MS^3^	0.5 µg/mL	0.5 µg/mL	110.5 ± 7.0	[59]
**(a) Eslicarbazepine acetate** **(b) Eslicarbazepine** **(c) Oxcarbazepine** **(d) (R)-licarbazepine**	Plasma	(a) and (c) 50 μL (b) and (d) 200 μL	PP (50% acetonitrile for eslicarbazepine acetate and oxcarbazepine; 100% acetonitrile for eslicarbazepine and (R)-licarbazepine)	LC-MS/MS	n.s.	n.s.	n.s.	[60]
Zonisamide	(a) Blood (b) Plasma	(a) 50 μL (b) 30 μL	(a) DBS (b) DPS	UHPLC–MS/MS	n.s.	(a) 0.125 µg/mL (b) 0.250 µg/mL	n.s.	[61]
Valproic Acid	Blood	200 µL	PP (acetonitrile)	LC-MS/MS	2 μg/mL	5 μg/mL	n.s.	[62]
Lacosamide	Plasma	100 µL	ODS	UHPLC-DAD	n.s.	0.25 µg/mL	96.6–106.2	[63]
Valproic Acid	Blood	100 µL	LLE (MTBE, TMSDM, and methanol)	GC-MS	1 µg/mL	n.s.	86.7–91.6	[64]
Gabapentin	(a) Serum (b) Plasma	n.s.	PP (75% methanol in Milli-Q water (*v*/*v*))	ID-LC-MS/MS	n.s.	n.s.	99–108	[65]
Topiramate	(a) Serum (b) Plasma	n.s.	PP (75% methanol in Milli-Q water (*v*/*v*))	ID-LC-MS/MS	0.0239 μg/mL	n.s.	95–102	[65]
(a) Carbamazepine (b) Oxcarbazepine	Plasma	n.s.	IT-SPME	MS	(a) 0.0002 µg/mL (b) 0.00025 µg/mL	(a) 0.00008 µg/mL (b) 0.0001 µg/mL	(a) 102.4–117.7 (b) 90.7–104.8	[33]
Levetiracetam	Plasma	40 μL	PP (protein precipitation solution)	UPLC-MS/MS	n.s.	0.1 µg/mL	97.4–101.1	[66]
Carbamazepine	Blood	15 μL	MI-IPN (DBS)	CE	n.s.	4 µg/mL	89.7–94.7	[67]
Levetiracetam	Serum	n.s.	n.s.	(a) HPLC-UV (b) IT	n.s.	n.s.	n.s.	[68]
Brivaracetam	Serum	n.s.	n.s.	LC-MS/MS	0.02 µg/mL	0.1 µg/mL	n.s.	[69]
(a) Carbamazepine (b) Carbamazepine-epoxide	Plasma	2 mL	PP (dichloromethane)	ICA	(a) 0.25 ng/mL (b) 1 ng/mL	n.s.	(a) 89.0–95.2 (b) 89.1–94.6	[70]
Padsenovil	(a) Blood (b) Plasma	n.s.	(a) VAMS (b) n.s.	n.s.	n.s.	n.s.	n.s.	[71]
Levetiracetam	Plasma	n.s.	n.s.	LC-MS/MS	n.s.	n.s.	n.s.	[72]
(a) Lamotrigine (b) Levetiracetam (c) Carbamazepine (d) Carbamazepine-epoxide (e) Topiramate (f) Valproic acid (g) Zonisamide (h) Oxcarbazepine	Plasma	n.s.	(a) n.s. (b) DBS	LC-MS	n.s.	(a) 0.1 µg/mL (b) 1.8 µg/mL (c) 0.7 µg/mL (d) 0.1 µg/mL (e) 1.6 µg/mL (f) 13.1 µg/mL (g) 1.0 µg/mL (h) 0.1 µg/mL	n.s.	[73]
Lamotrigine	Plasma	50 μL	SPE	LC-MS/MS	n.s.	0.2 µg/mL	93.8–98.6	[74]
Valproic Acid	Plasma	n.s.	SPE	UHPLC-MS/MS	n.s.	0.05 µg/mL	81.4–110	[75]
Valproic Acid	Serum	50 μL	PP (sulfuric acid, ether, and tetramethylammonium hydroxide)	HPLC-UV	0.4 µg/mL	1.0 µg/mL	91.6–97.4	[76]
(a) Carbamazepine (b) Carbamazepine-epoxide	Serum	100 μL	PP (acetonitrile)	UHPLC-MS/MS	n.s.	(a) 0.05 µg/mL (b) 0.01 µg/mL	74.7– 93.48	[77]
Lamotrigine	Serum	200 μL	PP (methanol/acetonitrile 1:1, *v*/*v*) and SPME	HPLC-DAD	n.s.	0.625 µg/mL	75.4–82.5	[78]
(a) Carbamazepine (b) Valproic acid (c) Phenobarbital (d) Phenytoin (e) Carbamazepine-epoxide	Blood	25 μL	DBS	LC-MS/MS	n.s.	(a) 2 µg/mL (b) 25 µg/mL (c) 1 µg/mL (d) 4 µg/mL (e) 0.25 µg/mL	(a) 53.24–71.96 (b) 50.37–67.03 (c) 51.41–70.99 (d) 50.75–68.25 (e) 51.21–70.79	[79]
Topiramate	Blood	n.s.	n.s.	LC-MS/MS	n.s.	n.s.	n.s.	[80]
**(a) Levetiracetam** **(b) Lamotrigine** **(c) Zonisamide** **(d) Topiramate** **(e) Carbamazepine** **(f) Phenytoin** **(g) Valproic Acid** **(h) Oxcarbazepine** **(i) 10,11-dihydro-** **10-hydroxycarbamazepine**	Plasma	50 μL	PP (acetonitrile)	LC-MS/MS	n.s.	(a) 0.005 µg/mL (b) 0.005 µg/mL (c) 0.01 µg/mL (d) 0.01 µg/mL (e) 0.005 µg/mL (f) 0.010 µg/mL (g) 0.05 µg/mL (h) 0.005 µg/mL (i) 0.005 µg/mL	(a) 93.7–102.9 (b) 95.6–103.8 (c) 93.7–105.7 (d) 100.1–109.3 (e) 98–104.4 (f) 98.6–104 (g) 68.9–73.9 (h) 98–104.6 (i) 93.7–103.6	[81]
Valproic Acid	Blood	n.s.	DBS	GC-MS	n.s.	n.s.	n.s.	[82]
Oxcarbazepine	Plasma	200 μL	PP (methanol)	HPLC–n.s.	n.s.	2 µg/mL	n.s.	[83]
Phenobarbital	Blood	0.1 g	PP (acetonitrile)	LC-HRMS	0.25 mg/kg	0.5 mg/kg	n.s.	[84]
Lamotrigine	Serum	n.s.	PP (methanol)	HPLC–n.s.	n.s.	n.s.	n.s.	[85]
Carbamazepine	Serum	n.s.	(a) MIP-SBSE (b) MIP-MSPE	HPLC-UV	n.s.	n.s.	n.s.	[86]
Carbamazepine	Serum	n.s.	n.s.	µ-BIS-LOV ELISA	n.s.	1.0 µg/L	93–110	[87]
Brivaracetam	Plasma	n.s.	n.s.	UHPLC-MS/MS	n.s.	n.s.	n.s.	[88]
Sultiame	(a) Blood (b) Plasma	100 μL	PP (methanol)	HPLC-MS/MS	n.s.	n.s.	(a) n.s. (b) 1.61–16.73	[89]
Phenobarbital	Plasma	n.s.	n.s.	LC-MS/MS	n.s.	n.s.	n.s.	[90]
(a) Oxcarbazepine (b) Licarbazepine	Plasma	n.s.	SPE	LC-HRMS		0.008 µg/mL	92.34–104.27	[91]
Zonisamide	Serum	n.s.	n.s.	(a) LTIA (b) HPLC-UV	n.s.	(a) 3 µg/mL (b) 0.5 µg/mL	n.s.	[92]
(a) Lamotrigine (b) Carbamazepine (c) Oxcarbazepine (d) Cabamazepine-Epoxide	Plasma	1 mL	PP (acetonitrile) and MSPE	HPLC-UV	(a) 0.01 µg/mL (b) 0.009 µg/mL (c) 0.007 µg/mL (d) 0.009 µg/mL	(a) 0.031 µg/mL (b) 0.027 µg/mL (c) 0.02 µg/mL (d) 0.028 µg/mL	(a) 86.8–101.8 (b) 82.5–99.2 (c) 80.6–98.9 (d) 78.7–98.5	[93]
Levetiracetam	Serum/Plasma	n.s.	n.s.	EI	n.s.	n.s.	n.s.	[94]
Perampanel	Plasma	n.s.	n.s.	LC-MS/MS	n.s.	n.s.	n.s.	[95]
Lamotrigine	Serum	n.s.	n.s.	TI	n.s.	n.s.	n.s.	[96]
(a) Fenfluramine (b) Norfenfluramine	Plasma	100 μL	PP (acetonitrile)	LC-MS/MS	n.s.	(a) 1.6 ng/mL (b) 0.82 ng/mL	n.s.	[97]
Perampanel	Plasma	n.s.	n.s.	HPLC-UV	n.s.	n.s.	n.s.	[98]
Primidone	Serum/Plasma	50 μL	PP (75% methanol *v*/*v*)	ID-LC-MS/MS	0.0620 µg/mL	n.s.	97–103	[99]
Carbamazepine	Serum/Plasma	50 μL	PP (75% methanol *v*/*v*)	ID-LC-MS/MS	0.115 µg/mL	n.s.	101–105	[99]
Phenobarbital	Serum/Plasma	n.s.	PP (75% methanol *v*/*v*)	ID-LC-MS/MS	0.697 µg/mL	n.s.	100–107	[99]
Zonisamide	Serum/Plasma	50 μL	PP(75% methanol *v*/*v*)	ID-LC-MS/MS	0.317 µg/mL	1.50 µg/mL	98–101	[99]
Levetiracetam	Plasma	40 μL	PP (acetonitrile)	UHPLC-MS/MS	n.s.	0.1 µg/mL	n.s.	[100]
Carbamazepine-epoxide	Serum/Plasma	50 μL	PP (75% methanol *v*/*v*)	ID-LC-MS/MS	0.0111 µg/mL	n.s.	94–105	[99]
Pregabalin	Serum	2 mL	PP (methanol)	SM	2.81 × 10^−8^ mol/L	8.5 × 10^−8^ mol/L	99.02–104.78	[101]
(a) Vigabatrin (b) Levetiracetam (c) Pregabalin (d) Gabapentin (e) Lamotrigine (f) Lacosamide (g) Zonisamide (h) Rufinamide (i) Topiramate (j) Oxcarbazepine (k) Carbazepine	Serum	150 μL	PP (acetonitrile)	LC-MS/MS	n.s.	n.s.	>93	[102]

µ-BIS-LOV ELISA: micro-bead injection spectroscopy lab on valve enzyme-linked immunosorbent assay; 2D-LC-UV: 2-dimension liquid chromatography with ultraviolet spectroscopy; BioSPME: bioanalytical solid-phase microextraction; CE: capillary electrophoresis; CE-DAD: capillary electrophoresis coupled with diode array detection; CF-UF: centrifugal ultrafiltration; DAD: diode array detector; DBSs: dried blood spots; DPSs: dried plasma spots; EI: enzyme immunoassay; EME: electromembrane extraction; GC-FID: gas chromatography coupled with a flame ionization detector; GC-MS: gas chromatography coupled with mass spectrometry; HCLC-UV-PDA: heart-cutting liquid chromatography with ultraviolet spectroscopy and photodiode array detection; HPLC-DAD: high-performance liquid chromatography with diode array detection; HPLC-UV: high-performance liquid chromatography with ultraviolet spectroscopy; ICA: immunochromatographic assay; ID-LC-MS/MS: isotope dilution liquid chromatography-tandem mass spectrometry; IT: immunoassay test; IT-SPME: in-tube solid-phase microextraction; LC-HRMS: liquid chromatography coupled with high-resolution mass spectrometry; LC-MS: liquid chromatography mass spectrometry; LC-MS^3^: liquid chromatography tandem mass spectrometry cubed; LC-MS/MS: liquid chromatography tandem mass spectrometry; LC-TOF-MS: liquid chromatography time-of-flight mass spectrometry; LLE: liquid–liquid extraction; LTIA: latex particle-enhanced turbidimetric immunoassay; MSPE: magnetic solid-phase extraction; MI-IPN: paper-based molecularly imprinted interpenetrating polymer network; MIP-MSPE: molecularly imprinted polymers–magnetic solid-phase extraction; MIP-SBSE: molecularly imprinted polymers–stir bar sorptive extraction; MS: mass spectrometry; NaOH: sodium hydroxide; ODS: organic deproteinization solution; PP: protein precipitation; RP-HPLC-UV: reverse-phase high-performance liquid chromatography with ultraviolet spectroscopy; SM: spectrofluorimetric method; SPR: surface plasmon resonance; TI: turbidimetric immunoassay; UA-EME: ultrasound-assisted electromembrane extraction; UHPLC-DAD: ultra-high-performance liquid chromatography coupled with diode array detection; UHPLC-MS/MS: ultra-high-performance liquid chromatography tandem mass spectrometry; VAMS: volumetric absorptive microsampling; VA-SHS-LPME: vortex-assisted switchable hydrophilicity solvent-based liquid phase microextraction.

**Table 3:** Sample pretreatment and determination of AEDs in urine samples.

Compounds	Volume	Extraction Method	Detection Method	LOD	LOQ	Recovery (%)	Reference
Oxcarbazepine	0.9 g	VA–SHS–LPME (N,N-dimethylbenzylamine, distilled water (1:1, *v*/*v*), and sodium hydroxide 1 M)	GC-MS	6.2 μg/kg	21 μg/kg	97–100	[32]
**(a) Carbamazepine** **(b) Carbamazepine-10,11-epoxide** **(c) Eslicarbazepine** **(d) Lamotrigine** **(e) Levetiracetam** **(f) Oxcarbazepine** (g) Phenytoin (h) 4-hydroxyphenytoin (i) Topiramate	25 µL	SPE (80:18:2 DCM/IPA/NH_4_OH)	LC-MS/MS	(a) 0.05 µg/mL (b) 0.05 µg/mL (c) 0.5 µg/mL (d) 500 ng/mL (e) 0.5 µg/mL (f) 0.5 µg/mL (g) 0.5 µg/mL (h) 0.5 µg/mL (i) 0.5 µg/mL	(a) 0.05 µg/mL (b) 0.05 µg/mL (c) 0.5 µg/mL (d) 500 ng/mL (e) 0.5 µg/mL (f) 0.5 µg/mL (g) 0.5 µg/mL (h) 0.5 µg/mL (i) 0.5 µg/mL	(a) 106.2 (b) 24.7 (c) 102.0 (d) 102.9 (e) 14.9 (f) 92.6 (g) 105.6 (h) 100.8 (i) 92.8	[107]
(a) Carbamazepine (b) Oxcarbazepine	n.s.	IT-SPME	MS	(a) 0.00008 µg/mL (b) 0.0001 µg/mL	(a) 0.0003 µg/mL (b) 0.0003 µg/mL	(a) 98.7–108.6 (b) 90.2–107.2	[33]
Valproic Acid	100 µL	LLE (MTBE, TMSDM, and methanol)	GC-MS	1 µg/mL	n.s.	86.2–98.0	[64]
Carbamazepine	n.s.	n.s.	Electrochemical Sensor	0.0018 μM	0.006 μM	99.0–100.7	[108]
(a) Levetiracetam (b) Lacosamide	1 mL	Filtration	UHPLC-DAD	(a) 0.026 µg/mL (b) 0.023 µg/mL	(a) 0.096 µg/mL (b) 0.093 µg/mL	98.69–101.87	[109]
Carbamazepine	n.s.	CEC	MHPLC-MS/MS	n.s.	n.s.	n.s.	[110]
Valproic Acid	n.s.	SPE	UHPLC-MS/MS	n.s.	0.2 µg/mL	74.1–112.3	[75]
Sultiame	n.s.	PP (methanol)	LC-MS/MS	n.s.	n.s.	1.61–16.73	[89]
Pregabalin	1 mL	n.s.	SM	2.81 × 10^−8^ mol/L	8.5 × 10^−8^ mol/L	99.08–104.96	[101]

CEC: capillary extraction column; GC-MS: gas chromatography coupled with mass spectrometry; IT-SPME: in-tube solid-phase microextraction; LC-MS/MS: liquid chromatography-tandem mass spectrometry; LLE: liquid–liquid extraction; MHPLC-MS/MS: multidimensional high-performance liquid chromatography tandem mass spectrometry; MS: mass spectrometry; SM: spectrofluorimetric method; SPE: solid-phase extraction; UHPLC-DAD: ultra-high-performance liquid chromatography coupled with diode array detection; VA-SHS-LPME: vortex-assisted switchable hydrophilicity solvent-based liquid phase microextraction.

**Table 4:** Sample pretreatment and determination of AEDs in oral fluid samples.

Compounds	Volume	Extraction Method	Detection Method	LOD	LOQ	Recovery (%)	Reference
Clonazepam	20 μL	n.s.	LC-MS	n.s.	n.s.	n.s.	[57]
Perampanel	10 μL	PSP (chloroform)	SERS	n.s.	n.s.	n.s.	[114]
(a) Carbamazepine (b) Carbamazepine-10,11-epoxide (c) S-licarbazepine (d) Lacosamide (e) Levetiracetam	100 μL	PP (dichloromethane)	HPLC-DAD	n.s.	n.s.	(a) 80.1–95.4 (b) 82.5–95.1 (c) 80.0–94.2 (d) 79.8 –93.9 (e) 78.0–94.4	[115]
Perampanel	(a) 50 µL (b) 1 mL	PP (acetonitrile)	LC-MS/MS	n.s.	n.s.	n.s.	[51]
Levetiracetam	40 μL	PP (protein precipitation solution)	UHPLC-MS/MS	n.s.	0.001 μg/mL	108.8–113.5	[66]
Rufinamide	250 μL	LLE (methanol, ammonium hydroxide solution pH 9.25, and dichloromethan(e))	HPLC-UV	0.05 μg/mL	0.25 μg/mL	87.2 ± 3.9	[52]
(a) Phenobarbital (b) Phenytoin (c) Carbamazepine (d) Carbamazepine-epoxide	50 μL	LLE (acidified methanol pH 5.5)	HPLC-DAD	0.05 μg/mL	0.1 μg/mL	(a) 43–57.1 (b) 48.1–64.4 (c) 38.7–49.1 (d) 38.8–55	[35]
(a) Carbamazepine (b) Carbamazepine-epoxide	n.s.	SPE	UHPLC-DAD	n.s.	n.s.	(a) 46.82–49.18 (b) 41.4–41.72	[116]
Levetiracetam	40 μL	PP (acetonitrile)	UHPLC-MS/MS	n.s.	0.1 µg/mL	n.s.	[100]

HPLC-DAD: high-performance liquid chromatography with diode array detection; HPLC-UV: high-performance liquid chromatography with ultraviolet spectroscopy; LC-MS: liquid chromatography coupled with mass spectrometry; LC-MS/MS: liquid chromatography tandem mass spectrometry; LLE: liquid–liquid extraction; PP: protein precipitation; PSP: phase separation process; SERS: surface-enhanced Raman scattering; UHPLC-DAD: ultra-high-performance liquid chromatography coupled with diode array detection; UHPLC-MS/MS: ultra-high-performance liquid chromatography tandem mass spectrometry.

## Data Availability

This systematic search was performed via the databases Web of Science and SCOPUS.
